# Humanized Biomimetic Nanovesicles for Neuron Targeting

**DOI:** 10.1002/advs.202101437

**Published:** 2021-08-11

**Authors:** Assaf Zinger, Caroline Cvetkovic, Manuela Sushnitha, Tomoyuki Naoi, Gherardo Baudo, Morgan Anderson, Arya Shetty, Nupur Basu, Jennifer Covello, Ennio Tasciotti, Moran Amit, Tongxin Xie, Francesca Taraballi, Robert Krencik

**Affiliations:** ^1^ Center for Musculoskeletal Regeneration Houston Methodist Research Institute Orthopedics and Sports Medicine Houston Methodist Hospital Houston TX 77030 USA; ^2^ Laboratory for Bioinspired Nano Engineering and Translational Therapeutics, Department of Chemical Engineering Technion−Israel Institute of Technology Haifa 3200003 Israel; ^3^ Center for Neuroregeneration Houston Methodist Research Institute Department of Neurosurgery Houston Methodist Hospital Houston TX 77030 USA; ^4^ Department of Bioengineering Rice University Houston TX 77030 USA; ^5^ Department of BioSciences Rice University Houston TX 77030 USA; ^6^ Department of Head and Neck Surgery The University of Texas MD Anderson Cancer Center Houston TX 77030 USA; ^7^ IRCCS San Raffaele Hospital Rome 00163 Italy

**Keywords:** biomimicry, human pluripotent stem cells, nanovesicles, neurons, organoids

## Abstract

Nanovesicles (NVs) are emerging as innovative, theranostic tools for cargo delivery. Recently, surface engineering of NVs with membrane proteins from specific cell types has been shown to improve the biocompatibility of NVs and enable the integration of functional attributes. However, this type of biomimetic approach has not yet been explored using human neural cells for applications within the nervous system. Here, this paper optimizes and validates the scalable and reproducible production of two types of neuron‐targeting NVs, each with a distinct lipid formulation backbone suited to potential therapeutic cargo, by integrating membrane proteins that are unbiasedly sourced from human pluripotent stem‐cell‐derived neurons. The results establish that both endogenous and genetically engineered cell‐derived proteins effectively transfer to NVs without disruption of their physicochemical properties. NVs with neuron‐derived membrane proteins exhibit enhanced neuronal association and uptake compared to bare NVs. Viability of 3D neural sphere cultures is not disrupted by treatment, which verifies the utility of organoid‐based approaches as NV testing platforms. Finally, these results confirm cellular association and uptake of the biomimetic humanized NVs to neurons within rodent cranial nerves. In summary, the customizable NVs reported here enable next‐generation functionalized theranostics aimed to promote neuroregeneration.

## Graphical abstract

Membrane proteins are extracted from either human pluripotent stem cells or differentiated neurons and combined with lipids to synthesize biomimetic nanovesicles (NVs). Neural targeting of NVs can be evaluated both in vitro using monolayer cultures and 3D organoids and in vivo by direct injection of NVs into murine trigeminal ganglions.

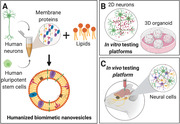

## 1 Introduction

Restoration of neural function after traumatic injury, neurodegeneration, or neuroinflammation is currently hindered by a lack of effective and clinically practicable biotechnologies for precise, cell‐targeted therapies or diagnostics. As such, there remains a need for biotechnological breakthroughs that can enhance and sustain the delivery of therapeutic cargos (e.g., genetic material and chemical compounds using nanomaterials),^[^ ^1^ ^]^ while also mimicking the microenvironment of the brain to avoid foreign body response.^[^ ^2^ ^]^ One promising pathway is the utilization of nanotechnologies inspired by nature, more commonly referred to as bio‐inspired or biomimetic tools. By mimicking the composition and biological functions of the cells in our body, biomimetic tools avoid potential side effects that occur from systemic administration of potential therapeutics or imaging tools, such as the inflammation that can occur when using viral‐based delivery approaches.^[^ ^3^, ^4^ ^]^ Thus, they offer the opportunity to gain insight into potentially safer and more tractable methodologies.^[^ ^5^ ^]^ For example, cell‐derived exosomes are promising drug delivery systems as they are one mechanism for natural extracellular communication.^[^ ^6^, ^7^, ^8^, ^9^, ^10^ ^]^ Still, new approaches are needed, given that the complexity and variability of biomimetic tools from cellular sources appropriate for the nervous system reduces their potential for scalable precision medicine.^[^ ^11^ ^]^

Functionalized nanoparticles (NPs) have high potential as well‐defined carriers for the selective and targeted delivery of therapeutic cargo to neural cells due to their size scale.^[^ ^12^, ^13^ ^]^ For example, NPs have been used for the functional delivery of drugs to the rodent brain in multiple pathologies.^[^ ^14^, ^15^, ^16^ ^]^ Various surface modifications, such as coupling targeting peptides^[^ ^17^ ^]^ or antibodies^[^ ^18^ ^]^ to NPs or modifying surface charge for selective neuron‐specific targeting,^[^ ^19^ ^]^ have been employed to increase targeting efficacy. Alternatively to NPs, exosome‐like lipid nanovesicles (NVs) have been used as both contrast agents and drug delivery vehicles to the brain while mimicking neural cellular communication.^[^ ^20^, ^21^ ^]^ However, standardized protocols for the storage and characterization of NVs have yet to be fully established,^[^ ^22^, ^23^ ^]^ while the low yield from biological sample sources^[^ ^24^ ^]^ reduces the potential of translating NVs to clinical applications. Here, we sought to devise and optimize enhancements to existing lipid NVs using a well‐defined and scalable cell source.

Previously, we endeavored to achieve enhanced bioactivity targeted to specific cell types by developing innovative hybrid biomimetic NVs that took advantage of specific cell types (e.g., native cellular targeting moieties) and synthetic NPs (e.g., ease of fabrication, scalability, and reproducibility) while bridging the gaps in therapeutic translation.^[^ ^5^, ^23^ ^]^ In particular, we demonstrated that the incorporation of leukocyte‐derived plasma membrane proteins into the phospholipid bilayer of NVs enables immune system avoidance and association with inflamed endothelial cells while delivering a therapeutic payload.^[^ ^25^ ^]^ We also determined the integration location and orientation of these membrane proteins on the NV lipid membranes and revealed an equal distribution of the cytoplasmic and exoplasmic domains on one representative leukocyte‐derived membrane protein, CD11b.^[^ ^26^ ^]^ Moreover, the biomimetic properties of these NVs resulted from the transfer of cellular adhesion proteins to the surface of NVs, which then mediated protein–protein interactions with target cells.^[^ ^27^ ^]^ Given that cellular interactions of neurons are in part attributed to cell–cell binding of adhesion proteins at the cell membrane surface, a similar approach for targeting neural cell types holds promising potential.^[^ ^28^, ^29^ ^]^ Nonetheless, testing of this approach with human neural cells (in order to aid in potential clinical translation) has been hampered by the lack of pure cell sources for reproducible and scalable production. Recent advances in the differentiation of human pluripotent stem cells (hPSC) into specific neural cell types^[^ ^30^, ^31^ ^]^ may enable the generation of biomimetic NVs and experimental testing platforms which, compared to platforms using rodent‐derived neural cells, would be less likely to induce an immunogenic reaction in humans and thus more appropriate for clinical translation.

Based on this premise, we have developed and defined a new class of biomimetic human neural NVs (a.k.a. neurosomes) using a reproducible and scalable protein source from a pure population of rapidly derived hPSC‐derived excitatory cortical neurons (iNeurons). Specifically, we used a bottom‐up microfluidic‐based synthesis method to bioengineer our novel NVs by combining phospholipids with the membrane proteins from iNeurons. We found that incorporation of neuron‐derived membrane proteins does not affect the physicochemical properties of NVs and, in fact, enhances their uptake into cultured neurons. We further confirmed proof‐of‐principle cellular targeting efficacy both in vitro and in vivo using sphere (a.k.a. organoid) cultures and direct administration to the rodent trigeminal ganglion, respectively.^[^ ^32^ ^]^ These studies advance the current paradigm of NV bioengineering for improved cellular targeting within the nervous system.

## 2 Results

### 2.1 Preparation of Cell Source for Membrane Proteins and Workflow Scheme

**Figure 1:**
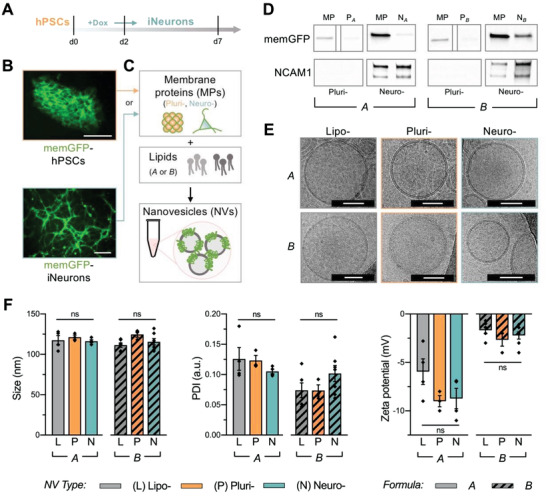
Physiochemical and biomimetic characterization of neural biomimetic NVs. A) iNeurons were directly generated from a genetically engineered human pluripotent stem cell (hPSC) line containing a doxycycline (dox)‐inducible neurogenin 2 (ngn2) transgene. A pure population was obtained within 7 days of differentiation. B) A stable membrane‐bound green fluorescent protein (memGFP) transgene was incorporated into the hPSC line to track protein carry‐over. Scale: 100 µm. C) A microfluidic approach was utilized for the synthesis of neural biomimetic NVs with cell‐specific membrane proteins and two different lipid formulations (i.e., A and B). Three NV groups were fabricated using each lipid formulation: “liposomes” (lipo‐, *L*) containing no protein, “plurisomes” (pluri‐, *P*) containing hPSC‐derived proteins, and “neurosomes” (neuro‐, *N*) containing iNeuron‐derived proteins. D) Immunoblotting revealed the transfer of mem‐GFP in plurisomes and neurosomes (NVs originating from hPSCs and iNeurons, respectively) as well as the transfer of neuronal membrane protein (MP) marker NCAM1 in neurosomes of both formulations. (Bands are replicated from Figure S1E (Supporting Information), with dividing lines indicating splicing from original image.) E) Cryo‐TEM images illustrated that all NV formulations had similar lipid bilayer morphologies containing a spherical bilayer structure. Scale: 50 nm. F) Physiochemical properties including NV size, PDI, and zeta potential were assessed. Though neither NV size nor PDI were significantly altered between NVs of different lipid formulations, NVs from lipid formulation B displayed a less negative zeta potential (*n* = 3–7 independent NV batches per group; see Figure S1F in the Supporting Information). For Figure 1F, results are shown as mean ± SEM. One‐way ANOVA followed by Tukey's multiple comparison test was used to determine statistical probabilities between NVs of different protein sources within the same formulation (A or B), with *p < 0.05.

First, we generated a pure population of neurons by directly inducing a genetically engineered hPSC line containing a doxycycline (dox)‐inducible neurogenin 2 (ngn2) transgene (**Figure** **1**A), as previously described in our established protocols,^[^ ^30^, ^31^ ^]^ to serve as a membrane protein source for functionalization of NVs. These hPSCs were directly induced into a uniform population of cortical glutamatergic excitatory neurons (iNeurons) with distinctive neuronal morphology by seven days of induction in a neural‐supportive medium. To determine whether bioengineered proteins can be produced in cells and transferred to NVs, a stable membrane‐bound green fluorescent protein (memGFP) transgene was incorporated via lentiviral delivery into hPSCs (Figure S1A, Supporting Information), which exhibited sustained memGFP expression during the differentiation process (Figure 1B). Proteins from the membranes of differentiated iNeurons and the parental hPSCs were extracted (Figure S1B, Supporting Information) for integration into NV lipid bilayers in order to generate two groups of biomimetic NVs: “neurosomes” (neuro‐, N) and “plurisomes” (pluri‐, P). As a control group, we prepared “liposomes” (lipo‐, L) (i.e., NVs without incorporated membrane proteins) for comparison.

### 2.2 Synthesis and Characterization of Biomimetic Humanized Neural Nanovesicles

We next utilized the NanoAssembler Benchtop system and a previously optimized, microfluidic assembly protocol^[^ ^23^, ^33^, ^34^ ^]^ to generate biomimetic NVs. In particular, cell‐derived membrane proteins were combined with phosphocholine‐based phospholipids and cholesterol at a 1:100 (w/w) ratio (Figure 1C). Next, two distinct lipid formulations were tested to enable the encapsulation of different potential therapeutic cargo (Table S1, Supporting Information). Lipid formulation A (i.e., L_A_, N_A_, and P_A_) was designed for the delivery of either proteins^[^ ^35^ ^]^ or small hydrophobic^[^ ^27^, ^34^, ^35^ ^]^ or hydrophilic drugs.^[^ ^36^ ^]^ It consisted of neutral lipids 1,2‐dipalmitoyl‐*sn*‐glycero‐3‐phosphocholine (DPPC), 1,2‐dioleoyl‐*sn*‐glycero‐3‐phosphocholine (DOPC), and cholesterol. Lipid formulation B (i.e., L_B_, N_B_, and P_B_) was designed to deliver genetic cargo (e.g., miRNA, mRNA, and siRNA)^[^ ^37^, ^38^ ^]^ and consisted of ionizable lipid 1,2‐distearoyl‐3‐dimethylammonium‐propane (16:0 DAP), 1,2‐distearoyl‐*sn*‐glycero‐3‐phosphoethanolamine N‐[carboxy(polyethylene glycol)‐2000] (DSPE‐PEG), and cholesterol. To demonstrate the potential delivery properties of these formulations, we encapsulated the glucocorticoid dexamethasone (Dex) and mRNA in liposomes generated with lipid formulations A and B, respectively. Release profiles of Dex and mRNA were assessed over 72 h at 37 °C (Figure S1C, Supporting Information). Consistent with previous demonstrations,^[^ ^25^ ^]^ we determined over ≈80% of encapsulated Dex to be released after 4 h from L_A_ NVs. In L_B_ NVs, less than 1% of the encapsulated mRNA was released after 72 h. This slow release has been demonstrated to be a result of the electrostatic connection between the charged phospholipids and the mRNA, which is released in the endosome at acidic pH.^[^ ^39^ ^]^

Next, we determined the extent to which membrane proteins were transferred into the biomimetic NVs. Coomassie blue staining after sodium dodecyl sulfate‐polyacrylamide gel electrophoresis (SDS‐PAGE) separation confirmed the successful transfer of proteins (Figure S1D, Supporting Information). The presence of transgene‐derived memGFP was observed solely in NVs integrated with cell‐derived proteins, as evidenced by immunoblotting (Figure 1D; Figure S1E, Supporting Information). NCAM1 (a known neural‐restricted protein involved in cell–cell interactions) was used as an indicator of endogenous cell‐type‐specific protein carry‐over. As expected, NCAM1 was present in protein fractions of neurosomes originating from iNeurons (e.g., N_A_ and N_B_), but not in plurisomes originating from hPSCs (e.g., P_A_ and P_B_). The same NCAM1 trend was observed among both lipid formulations A and B. Hence, we confirmed transfer of both genetically engineered membrane proteins as well as endogenous proteins into NVs using human cells.

To determine whether the incorporation of human proteins would disrupt defining features of NVs, we characterized the physicochemical properties and reproducibility of the distinct formulations. First, visualization using cryogenic transmission electron microscopy (cryo‐TEM) revealed preservation of a spherical bilayer structure and spherical shape, irrespective of alterations in the lipid formulation or cellular membrane protein source (Figure 1E). In addition, we verified homogeneity of size, polydispersity index (PDI), and surface charge (zeta potential) between and among formulations (Figure 1F; Figure S1F, Supporting Information). Neither the mean diameter nor the PDI was significantly altered by modification of membrane protein content (i.e., lipo‐ vs neuro‐ vs pluri‐) and the lipid formulation (i.e., A vs B). However, distinct lipid compositions produced significant differences in zeta potential between formulations A and B, but not among groups of the same formulation. Specifically, liposomes, plurisomes, and neurosomes in formulation B demonstrated average decreases of 3.7‐fold, 3.4‐fold, and 3.9‐fold, respectively, compared to formulation A. Overall, the characterization of these physiochemical properties verified the ability to reliably produce uniform NVs using these distinct lipid compositions and protein sources.

### 2.3 Confirmation of NV Association with Human Pluripotent Stem‐Cell‐Derived Neurons

**Figure 2:**
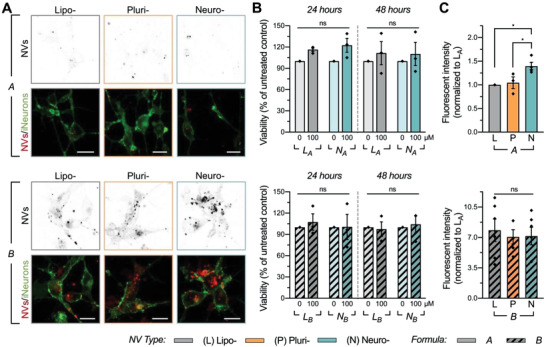
Association and cytotoxicity testing of NVs on neuronal monolayer cultures. A) Confocal microscopy was utilized to qualitatively assess NV‐iNeuron association. Images demonstrate 24 h of incubation with 750 × 10^−6^ m rhodamine labeled NVs. Scale: 10 µm. B) Neither lipid formulation A (top) nor B (bottom) of neurosomes and liposomes resulted in significant cytotoxicity at 100 × 10^−6^ m up to 48 h post‐treatment compared to untreated cells, as determined by a viability assay (*n* = 3–4 independent batches of cells per group; see Figure S2B–D in the Supporting Information). C) iNeurons were incubated with rhodamine labeled NVs for 24 h to quantitatively confirm association of liposomes, plurisomes, and neurosomes of formulations A (top) and B (bottom) with high throughput fluorescence‐activated cell sorting (FACS). Data are presented as median fluorescent intensities normalized to L_A_ (*n* = 3–6 independent batches of cells and NVs). iNeurons exhibited significantly increased preferential association with N_A_ compared to L_A_ and P_A_ and overall higher association with NVs of lipid formulation B compared to A. For (B) and (C), results are shown as mean ± SEM. One‐way ANOVA followed by Tukey's multiple comparison test was used to determine statistical probabilities between concentrations of NVs within the same formulation and incubation time in viability experiments B), and between NVs of different protein sources within the same formulation in FACS experiments C), with *p < 0.05.

We first tested the ability of NVs to associate with neural cells in vitro. iNeurons were differentiated in monolayers and incubated with rhodamine labeled NVs for 24 h. NV association was confirmed using confocal microscopy (**Figure** **2**A; Figure S2A, Supporting Information). Notably, we qualitatively observed higher levels of cellular memGFP and NV colocalization in formulation B compared to formulation A. However, quantitative evaluation of monolayer cultures was challenged by technical concerns including association of NVs with underlying basement membrane substrate.

In order to confidently measure neuronal association at the quantitative level, we first evaluated NV cytotoxicity at various doses to determine their in vitro safety profiles. memGFP‐iNeurons were incubated with NVs for 24–72 h at concentrations ranging from 100 × 10^−6^ to 1 × 10^−3^ m. Neither lipid formulation of neurosomes nor liposomes produced significant neurotoxicity at 100 × 10^−6^ m for up to 48 h of treatment time compared to untreated cells, as determined by a cellular viability assay (Figure 2B). However, an increase in concentration surpassing 500 × 10^−6^ m in formulation B led to decreased viability over time (Figure S2B–D, Supporting Information). Based on this, a 24 h treatment of 100 × 10^−6^ m NVs was deemed optimal for subsequent testing.

Next, to validate selective cellular association of NVs, iNeurons were incubated with 100 × 10^−6^ m rhodamine labeled NVs for 24 h, after which iNeurons underwent a thorough washing to remove all nonassociated and noninternalized NVs before and after dissociation. Quantitative evaluation of iNeuron‐NV association was assessed using high throughput fluorescence‐activated cell sorting (FACS). Untreated cells were used to set the cell population gate from the scatter population (Figure S3A,B, Supporting Information). The median fluorescence intensity of each sample was normalized to the median fluorescence intensity of the rhodamine signal from L_A_, and then further normalized to the difference in fluorescence intensity between NV groups (Figure S3C, Supporting Information). Similarly, as observed with confocal microscopy, iNeurons exhibited a trend of preferential association with plurisomes and a significantly higher preferential association with neurosomes compared to liposomes from formulation A (1.4 ± 0.08‐fold increase in fluorescent intensity of N_A_ compared to L_A_; *p* = 0.01) (Figure 2C; Figure S3D, Supporting Information). Moreover, the association of iNeurons with neurosomes was significantly higher than with plurisomes from formulation A (*p* = 0.03). Overall, cellular association was observed to be higher with NVs generated with lipid formulation B (7.83 ± 1.29‐, 7.07 ± 0.83‐, and 7.16 ± 1.01‐fold increases in fluorescent intensity of L_B_, P_B_, and N_B_ compared to fluorescent intensity of L_A_), although there were no significant differences between NVs of different protein sources.

To assess the specificity of neuronal targeting, we first performed association tests on other human neural cell types in vitro. Mature hPSC‐derived human astrocytes^[^ ^40^ ^]^ and immortalized human microglia (HMC3 line) were incubated with 100 × 10^−6^ m rhodamine labeled NVs from both formulations A and B for 24 h. After thorough washing, cells were dissociated and evaluated for NV association using FACS as described above for iNeurons. No significant increase was observed with regards to cellular association of either N_A_ or P_A_ compared to L_A_ in either astrocytes (Figure S4A, Supporting Information) or microglia (Figure S4B,C, Supporting Information). Notably, in microglia we measured a significantly lower association of both N_A_ and P_A_ compared to L_A_ (0.54 ± 0.1‐ and 0.6 ± 0.06‐fold decreases, respectively). In both cell types, a higher overall cellular association was observed with NVs generated with lipid formulation B, similarly as observed in iNeurons (Figure 2C); however, no significant differences in cellular association were measured between L_B_, P_B_, and N_B_ compared to L_A_.

Finally, we investigated a mechanism by which the NVs associate with human neurons. The potential of neural‐restricted adhesion molecule NCAM1 as a mechanism for enhanced neuronal association was examined by fabricating NVs integrated with only human recombinant NCAM1 protein (in place of total cell‐derived membrane protein) into NV lipid bilayers. Following successful fabrication, we evaluated association of NCAM1_A_ and NCAM1_B_ (i.e., NCAM1 NVs fabricated with lipid formulations A and B, respectively) with iNeuron monolayers using FACS. Correlating to our previous results, we observed a significant ≈5.2‐fold higher association of iNeurons with NCAM1_B_ compared to NCAM1_A_ (Figure S4D, Supporting Information). Notably, both NCAM1_A_ and NCAM1_B_ (1.34 ± 0.1‐ and 7.0 ± 1.7‐fold increases compared to fluorescent intensity of L_A_) demonstrated similar association to iNeurons as did N_A_ and N_B_ (1.4±0.08‐ and 7.16±1.01‐fold increases), respectively (Figure 2C), suggesting that NCAM1 could be one of the membrane proteins affecting neuronal association of NVs.

### 2.4 Testing Humanized NV Association within Human Organoids and Animal Models

Following the observations using FACS, wherein N_A_ exhibited increased selective targeting to iNeurons compared to both L_A_ and formulation B, we aimed to further validate these results using a 3D in vitro system.^[^ ^30^, ^31^ ^]^ We expected that 3D culture systems (a.k.a. spheroids or organoids) would serve as more physiologically relevant and predictive models than monolayer cultures due to their retention of extracellular matrix components and induction of cellular maturation that more closely mimics the native extracellular environment.^[^ ^41^ ^]^

**Figure 3:**
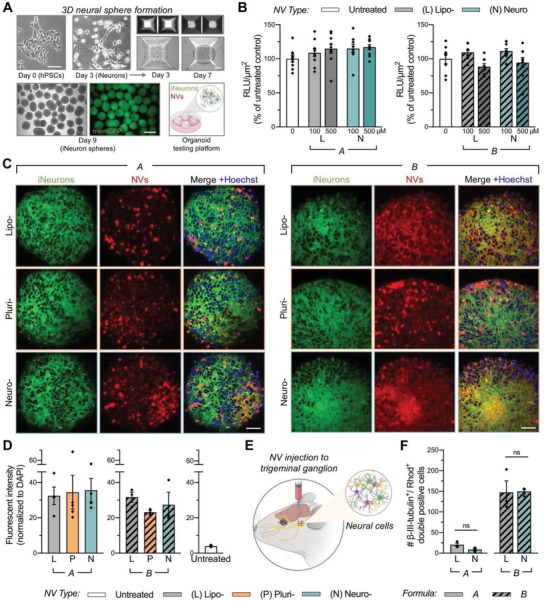
Cytotoxicity and association of humanized NVs within 3D model systems. A) Large‐scale production of 3D neural spheres was achieved by culturing differentiated, hPSC‐derived iNeurons in microwell plates. These organoid‐based spheres were utilized as a humanized testing platform for NVs. Scale: 100 µm (top) and 500 µm (bottom). B) Cell viability of iNeurons cultured in 3D spheres and treated with NVs from lipid formulation A (left) or B (right) at 100 or 500 × 10^−6^ m for 24 h, as determined by a CellTiter‐Glo 3D Assay. Outliers were identified and removed using the ROUT method (based on maximum false discovery rate *Q* = 1%) in GraphPad Prism. Relative luminescence units (RLU) were normalized to sphere cross‐sectional areas and untreated control spheres (*n* = 8–10 spheres per group). In subsequent experiments, 3D spheres were treated with 500 × 10^−6^ m NVs from both formulations A and B. C) Qualitative analysis of maximum projections images from z‐stacks demonstrated association of rhodamine labeled NVs (from both formulations A and B) with iNeurons in 3D spheres. Scale: 50 µm. D) NV association was quantified by assessing the raw integrated density of the rhodamine signal in maximum projection images, normalized to nuclear signal within each sphere (*n* = 3–5 spheres per group). E) N_B_ and L_B_ were administrated to the left trigeminal ganglion of C57BL/6 mice. Tissue samples were collected and processed for FACS analysis 24 h post‐treatment. F) FACS analysis indicated similar levels of association between neurons and NVs (liposomes and neurosomes) for both lipid formulations A and B, as assessed by double‐positive signal of rhodamine (NVs) with fluorescently labeled beta‐III tubulin (*n* = 3 mice per group of NVs). For (B), (D), and (F), results are shown as mean ± SEM. For (D) and (F), significance was determined using a two‐tailed unpaired t‐test between neurosomes and liposomes in formulations A and B for in vivo FACS experiments, with *p < 0.05.

Organoid‐based spheres were generated using our previously optimized protocol^[^ ^31^ ^]^ by culturing hPSC‐derived iNeurons in microwell plates, yielding spheres of uniform shape and size (**Figure** **3**A). The effects of NV treatment on the cellular viability of this model were first determined through a 3D CellTiter‐Glo viability assay, in which spheres were incubated with NVs for 24 h at concentrations ranging from 100 to 500 × 10^−6^ m, after neuronal maturation (Figure 3B). The results suggests that sphere cultures can tolerate NV treatment better than monolayer cultures can, similar to what has previously been reported with liver cancer spheres.^[^ ^42^ ^]^ Subsequently, iNeuron spheres were incubated with 500 × 10^−6^ m NVs for 24 h and imaged on a confocal microscope (Figure 3C). To measure colocalization, rhodamine fluorescent intensity from maximum projections of z‐stack images was normalized to sphere size. NVs of both formulations effectively associated with iNeurons in spheres (Figure 3D); however, signal intensity varied between spheres within and among groups.

Lastly, we assessed the potential of ex vivo slice cultures as an alternative evaluation platform for NV association. Murine brain slices were treated with 500 × 10^−6^ m NVs for 24 h and imaged on a confocal microscope. Immunofluorescent staining for neuron‐specific *β*‐III‐tubulin and glial‐specific fibrillary acidic protein (GFAP) qualitatively suggested higher association of N_A_ and N_B_ with neuronal cell bodies than with astrocytes (Figure S5, Supporting Information). Given that astrocytes within brain slices can become reactive, which affects their potential ability to associate with NVs,^[^ ^43^ ^]^ further study of biocompatibility and cellular association was conducted by locally injecting fluorescent NVs (liposomes and neurosomes) to the left trigeminal ganglion of C57BL/6J mice (Figure 3E; Figure S6A, Supporting Information), similarly as performed in our previously described study.^[^ ^37^ ^]^ 24 h postinjection, the left trigeminal ganglion was collected and FACS analysis was performed to determine the association of L_A_, N_A_, L_B_, and N_B_ NVs with neurons and astrocytes in vivo (Figure S6B–D, Supporting Information). When considering colocalization of rhodamine (i.e., NV marker) and fluorescently labeled beta‐III tubulin (i.e., a cell‐restricted neuronal marker), no significant differences in neuronal cellular association levels between liposome‐ and neurosome‐treated groups of the same formulation (an average of 20.3 ± 3.9 and 8.7 ± 3.0 double‐positive events in L_A_‐ and N_A_‐treated groups, and an average of 147.3 ± 28.1 and 149.7 ± 5.8 double‐positive events in L_B_‐ and N_B_‐treated groups, respectively) were found (Figure 3F). Similarly, no significant differences between liposome‐ and neurosome‐treated groups of the same formulation were observed in cellular association of NVs with astrocytes (an average of 26.0 ± 6.1 and 11.7 ± 3.0 double‐positive events in L_A_‐ and N_A_‐treated groups, and an average of 163.7 ± 37.8 and 149.4 ± 11.7 double‐positive events in L_B_‐ and N_B_‐treated groups, respectively) (Figure S6E, Supporting Information). Corresponding with the significantly higher neuronal association observed with formulation B compared to formulation A in vitro (Figure 2C), we observed a higher quantity of double‐positive events in both NV types of formulation B compared to formulation A, in neurons (Figure 3F) as well as astrocytes (Figure S6E, Supporting Information).

## 3 Discussion

Here, we developed and validated a multifunctional, biomimetic nanotechnology platform that not only holds promising potential to target various neural cell types, but also permits the ability to deliver diverse theranostic cargo. To the best of our knowledge, these humanized neural NVs are the first of their kind and represent a blueprint for the future development of NVs derived from other human cell sources and with different lipid formulations. By assessing the physiochemical and biological properties of the various synthesized neural NVs, and by testing association and viability upon organoid cultures, we established the broad potential of this biomimetic approach for use in disease contexts within the central and peripheral nervous systems or with experimental drug testing platforms.

The utilization of a consistent protein source from distinct stages of cellular differentiation, made possible by a transdifferentiation method^[^ ^44^, ^45^ ^]^ which rapidly and reproducibly generates a pure population of iNeurons through a single transgene induction method, was of considerable importance when synthesizing the neural NVs, particularly in order to incorporate both endogenous and engineered proteins in the NVs. In conjunction, the use of a microfluidic‐based synthesis method further enhanced the reproducibility of the NV formulations. With a stable protein source and reproducible fabrication method in hand, we expanded the multifunctional capabilities of the NVs for the delivery of versatile therapeutic cargo by testing the synthesis process using two different lipid backbones. The use of both nonneural protein containing NVs (i.e., plurisomes) and NVs with no protein at all (i.e., liposomes) enabled us to correlate and attribute the targeting trends observed in vitro and in vivo back to the absence or presence of the distinct proteins’ sources. These results also suggest that the absence or presence of PEG on the lipid backbone is an important additional component to account for, especially when considering the demonstrated ability of PEG to minimize cell–cell interactions in the bloodstream.^[^ ^46^ ^]^ Here, we modeled local injection of NVs and did not assess the ability to circulate in the blood nor to cross the blood–brain–barrier. Subsequent studies will be needed to evaluate the effect of PEG on NV blood circulation times.

Furthermore, the detection of both endogenous and exogenous membrane proteins on the synthesized NVs validated the successful carry‐over of operator‐desired proteins from the cell source. The implications of the membrane protein transfer underscore the potential applications of this approach for emerging targeted or therapeutic technologies as well as for protein–protein interaction studies.^[^ ^47^ ^]^ In the future, this customizable approach can be applied toward the engineering of NVs with cell‐restricted proteins or proteins from other nervous system cell types (i.e., astrocytes, oligodendrocytes, microglia, etc.) and can also be used to target specific membrane protein‐mediated cascades. Moreover, as we previously demonstrated,^[^ ^26^ ^]^ improved targeting can be achieved through modulation of the protein:lipid ratio, which is a key synthesis parameter of these NVs.

The inclusion of distinct membrane proteins using this fabrication method did not negatively impact the morphology, size, and homogeneity of the resulting bilayer NVs, especially when compared to their nonprotein containing counterparts (i.e., liposomes). However, differences were observed in the surface charge between the two lipid formulations (i.e., A vs B). While the successful integration of negatively charged proteins decreased the zeta potential of the NVs, this expected result was only pronounced in formulation A. Notably, the neutral surface charge measurements in formulation B was due to the presence of the long PEG‐2000 chains. This, in combination with the neuron membrane protein, resulted in increased zeta potential compared to NVs from formulation A. This near‐neutral zeta potential (i.e., surface charge) was also demonstrated by Xu et al. when using PLGA‐PEG NPs.^[^ ^48^ ^]^ Further, work by Kostarelos et al^[^ ^49^ ^]^ and others has shown that the addition of PEG chains can obstruct cellular uptake of liposomes in sphere cultures, and increase tissue penetration.^[^ ^50^ ^]^

In vitro screening of our innovative NVs demonstrated both their toxicity profile and association behavior to neural cells, with N_A_ exhibiting selective association compared to L_A_. Although this phenomenon was not observed using formulation B NVs, an overall higher association was noticed in this group compared to formulation A. Though we did not test these hypotheses in this study, the absence of this selective targeting may be explained through two key mechanisms mediated by the presence of the PEG‐2000 lipid in formulation B NVs. On the one hand, it is possible that the long‐chain length of the PEG‐2000 lipid hampers the direct protein–protein interactions of the NVs membrane protein with those on iNeuron membranes. On the other hand, the PEG‐2000 brush configuration on the NVs surface may provide stealth properties that prevent protein adsorption on their surface, thus mediating higher tissue penetration, as was reported by Xu et al.^[^ ^48^ ^]^

3D in vitro sphere cultures provide the opportunity to robustly assess NVs or drug distribution, toxicity, and overall efficacy in a relevant, scalable, and customizable microenvironment. In vivo studies with animals can be time‐consuming and costly.^[^ ^51^, ^52^ ^]^ As many assays or transplantations are not feasible in humans, especially in the human brain,^[^ ^53^ ^]^ reliable high‐throughput in vitro cellular models—which could permit the safe and controlled testing of dosage, exposure, and various cell types or organ systems in real time physiological or pathological conditions that are relevant to humans—are vital for preclinical experimentation.^[^ ^53^, ^54^ ^]^ As organoid‐based models can better recapitulate the physiological microenvironment, spatial complexity, and cellular organization and interactions compared to monolayer cultures,^[^ ^55^ ^]^ several others have employed 3D tissue cultures to characterize toxicity and pharmacokinetics in vitro. Recent examples of nonneural 3D platforms have included kidney organoids,^[^ ^56^ ^]^ intestinal organoids,^[^ ^57^ ^]^ colorectal^[^ ^51^ ^]^ and liver cancer spheroids^[^ ^42^ ^]^ as a means to examine physiochemical properties and accumulation of various metallic and polymer‐based NPs or other drug‐loaded vesicles. There remains a need for well‐defined and active neural sphere culture models that can be utilized as testing platforms for biomimetic, lipid‐based nanotechnologies that could be applicable toward the mature human nervous system. Nonetheless, despite multiple advantages, in vitro sphere cultures are somewhat limited with regards to in vivo predictive power. Moreover, they often lack mechanical forces or fluid flow, while the absence of vasculature prohibits direct translation to in vivo studies.^[^ ^50^ ^]^ Though animal models can sometimes be poorly indicative of human conditions and outcomes,^[^ ^58^ ^]^ they are undoubtedly useful and necessary for clinical translation of many nanotechnologies. Thus, we tested NV association with neural cells in vivo. Almost the same number of double‐positive cells were detected during the FACS analysis, with both treatment groups exhibiting higher association than that observed in the untreated mice. These findings support the association and uptake trends witnessed in vitro, though there remains a need for improvement of selective cell targeting.

The rapid, cost‐effective, standardizable one‐step process utilized here does not require chemical synthesis or solvent purification.^[^ ^23^, ^59^ ^]^ Moreover, the use of a pure population of hPSC‐derived neurons allows for a scalable source of cellular membrane proteins within a matter of days. These customizable and reproducible biomimetic strategies represent a paradigm shift in the design and engineering of neural‐specific NPs or NVs, enabling next‐generation technology platforms capable of effectively interfacing and interacting with complex biological systems.^[^ ^34^, ^60^, ^61^ ^]^ One example of potential neurotherapeutic applications of this system is the delivery of neural growth factors to promote outgrowth or synaptic connectivity. The sphere cultures can be used as preclinical screening platform to assess translational potential after injury ^[^ ^62^ ^]^ or during disease. Further improvements to this approach would strengthen the utility of this nanotechnology for specific applications. For example, the inclusion of an additional step of protein purification would remove the carry‐over of undesired membrane proteins which may affect the intended NV targeting and association. Moreover, adjusting the protein‐to‐lipid ratio (w/w) on the NVs could enhance the selective targeting, as recently demonstrated.^[^ ^26^ ^]^ Given the translational advantages and the fabrication tunability (i.e., both lipids and proteins) offered by this technology, these biomimetic NVs provide an innovative approach for the targeted delivery of needed therapeutic cargoes to neurological diseases.

## 4 Experimental Section

### Cell Culture

Human pluripotent stem cells (hPSCs) (line WTC11, Coriell #GM25256) were cultured in a pluripotent maintenance medium (hPSC medium) that consisted of TeSR‐E8 basal medium with supplements (STEMCELL Technologies) and 1× antibiotic–antimycotic (Gibco). For iNeuron experiments, cells were infected with a lentivirus to express a membrane GFP transgene (Addgene #22479) and manually purified by clone selection. At 80% confluency, hPSCs were either collected for membrane protein extraction or differentiated to iNeurons. Differentiation was prompted by exchanging basal hPSC medium with a neural‐supportive medium (NM) consisting of DMEM/F‐12 with GlutaMAX (Thermo Fisher), 0.5× N‐2 and 0.5X B‐27 supplements (Gibco), 2 mg mL^−1^ heparin (Sigma‐Aldrich), and 1× antibiotic–antimycotic, with the addition of 2 µg mL^−1^ doxycycline hydrochloride (Dox; Sigma‐Aldrich) for neuronal induction. Cells were maintained as a monolayer in NM+Dox for 2 days, treated with Accutase (Sigma‐Aldrich) for cell detachment, and then replated on Matrigel‐coated plates in the presence of Rho‐kinase inhibitor Y27632 (Tocris). On day 7, iNeurons were either collected for membrane protein extraction or treated with NVs for association studies. Alternatively, day 2 iNeurons were cultured in Aggrewell 800 24‐well microwell plates (STEMCELL Technologies) at a density of 2 × 10^6^ cells per microwell to generate spheres, as previously described.^[^ ^30^, ^31^ ^]^ After 2 days, spheres were gently removed from microwells and cultured in T75 flasks, with media changes every 3 days, until the time of experiment. For astrocyte experiments, human astrocytes (differentiated from the H9 [WA09] hPSC line, as previous described^[^ ^40^ ^]^) were maintained in NM with epidermal growth factor (EGF) and fibroblast growth factor‐2 (FGF2; 10 ng mL^−1^ each, Peprotech). For microglia experiments, the human microglial clone 3 (HMC3) cell line (CRL‐3304, ATCC) was maintained in NM with 5% heat‐inactivated fetal bovine serum (FBS, Gibco). At the time of experimentation, growth factors and serum were removed, and cells were cultured in monolayers and treated with NVs for association studies, as described below.

### Membrane Protein Extraction and Quantification

Membrane proteins were prepared from multiple independent batches of differentiation of hPSC‐derived iNeurons for neurosomes and multiple subsequent passages of hPSCs for plurisomes. From these, separate independent batches of each formulation of each NV type were prepared. Membrane proteins were extracted from live iNeurons and hPSCs using a ProteoExtract Native Membrane Protein Extraction Kit (Millipore Sigma) according to the manufacturer's protocol. Quantification of extracted membrane proteins was performed using a Pierce Rapid Gold BCA Protein Assay Kit (Fisher Scientific) according to the manufacturer's protocol. Absorbance was measured at 480 nm on a FLUOstar Omega microplate reader (BMG Labtech), and protein concentration was determined using a standard curve. Extracted membrane protein supernatants were stored with protease inhibitor at −80 °C until use.

### NV Synthesis and Purification

NVs were synthesized using a NanoAssemblr (Precision Nanosystems). Formulation A consisted of Dipalmitoylphosphatidylcholine (DPPC), 1,2‐dioleoyl‐*sn*‐glycero‐3‐phosphocholine (DOPC), and cholesterol (ovine wool, >98%) (4:3:3 molar ratio), while Formulation B was comprised of 16:0 1,2‐dipalmitoyl‐3‐dimethylammonium‐propane (DAP), DSPE‐PEG2000, and cholesterol (4.2:1:4.8 molar ratio) (all from Avanti Polar Lipids, Inc; see Table S1 in the Supporting Information). 1:100 (w/w) protein: lipid ratios were used for plurisome, neurosome, and NCAM1 NV formulations. The organic phase containing lipids was dissolved using 100% ethanol. The aqueous phase for formulation A consisted of 1× PBS alone (for liposomes) or 1× PBS with extracted membrane proteins (for plurisome and neurosomes) or with recombinant human NCAM1 protein (R&D Systems). The aqueous phase for formulation B consisted of 125 × 10^−3^ m sodium acetate buffer (pH = 5.2) alone (for liposomes) or 125 × 10^−3^ m sodium acetate buffer (pH = 5.2) with extracted membrane proteins (for plurisome and neurosomes). After preparing the two phases for each formulation, the NanoAssemblr microfluidic chip was first washed with ethanol and then with either 1× PBS or 125 × 10^−3^ m sodium acetate buffer (pH = 5.2), depending on which formulation was to be prepared next. The organic and aqueous phases were loaded into individual syringes, allowed to warm for 3 min on a heating block set at 50 °C, and then connected to the inlet ports of the chip. Particles were then synthesized using the following parameters for the machine: formulation A—total flow rate = 1 mL min^−1^, organic flow rate = 0.333 mL min^−1^, aqueous flow rate = 0.667 mL min^−1^, initial waste = 0.15 mL, final waste = 0.05 mL; formulation B—total flow rate = 1 mL min^−1^, organic flow rate = 0.350 mL min^−1^, aqueous flow rate = 0.650 mL min^−1^, initial waste = 0.15 mL, final waste = 0.05 mL. Synthesized particles were then dialyzed overnight using 1000 kDa Float‐A‐Lyzer G2 dialysis devices (Spectrum Labs) at 4 °C in 1× PBS (1:1000 v/v), with one buffer change after 1 h. After dialysis, particles were collected and filtered using 0.22 µm PVDF syringe filters (Fisher Scientific). Rhodamine labeled NVs were fabricated as described above with the addition of 0.005 mg of 1,2‐dioleoyl‐*sn*‐glycero‐3‐phosphoethanolamine‐N‐(lissamine rhodamine B sulfonyl) (ammonium salt) to the organic phase for every 1 × 10^−3^ m of lipids.

### Drug Release Studies

Dexamethasone (Dex) was encapsulated within liposomes from lipid formulation A (L_A_) and evaluated using high‐performance liquid chromatography (HPLC), as previously demonstrated.^[^ ^63^ ^]^ Measurements were performed on a Waters e2695 unit equipped with a UV/vis detector module UV/vis 2489 and a Phenomenex Luna (5 µm) C18 column, 250 × 4.6 mm. Separation was performed under an isocratic mobile phase of water: acetonitrile 70:30% (v/v). Samples were run under a 1 mL min^−1^ flow and absorbance was measured at 254 nm at 10 °C. The Dex release profile was measured by placing samples inside individual 1000 kDa Float‐A‐Lyzer dialysis devices (Spectrum Labs) in separate beakers for each time point (0, 1, 2, 4, 8, 24, 48, and 72 h). Beakers were filled with 500 mL of 1× PBS under agitation while maintaining a temperature of 37 °C. mRNA (CleanCap FLuc mRNA 5moU, TriLink BioTechnologies) was encapsulated within liposomes from lipid formulation B (L_B_). Briefly, mRNA encapsulation was measured using a TECAN plate reader at 480 nm excitation and 520 nm emission after 10 min incubation with Quanti‐iT Rybogreen‐iT. The mRNA release profile was measured by collecting samples from the tubes on specific time intervals (0, 1, 2, 4, 8, 24, 48, and 72 h) while comparing the encapsulated versus total mRNA. Total mRNA was measured by bursting the NVs using 2% Triton‐X100 followed by a 10 min incubation at 37 °C on a stirring plate. Free mRNA was measured without the addition of Triton‐X100, as previously demonstrated.^[^ ^64^ ^]^

### Membrane Protein Marker Detection

The presence of GFP and NCAM1 membrane proteins on the NVs was verified using Western blot after dialysis. Primary and secondary antibodies were then added to detect GFP and NCAM1. For GFP detection, Anti‐GFP (Green Fluorescent Protein) antibody (Chicken Antibodies, IgY Fraction) (GFP‐1010) 1:2500 diluted (Aves Labs) followed by incubation with Goat antichicken IgY H&L (HRP) (ab6877) 1:2000 diluted. For NCAM1 detection, Human/Mouse NCAM‐1/CD56 antibody (AF2408) 1:2500 diluted (R&D systems) followed by incubation with antigoat IgG‐HRP (HAF017) 1:2000 diluted. Gels were imaged using a Bio‐Rad imaging system.

### Cryo‐TEM of NVs

NVs solutions were vitrified and imaged at the Baylor College of Medicine Cryo‐Electron Microscopy Core Facility (Houston TX) as reported by Zinger et al.^[^ ^35^ ^]^ Briefly, Quantifoil R2/1, 200 Cu mesh Holey carbon grids were pretreated with airglow discharge for 45 s to make the carbon surface hydrophilic. In addition, Quantifoil R2/1 200 Cu +4 nm thin carbon grids were also glow discharged for 10 s to test the efficacy of the added layer of continuous carbon with the binding of the NVs. Vitrification was done using a Vitrobot Mark IV (FEI) operated at 18 °C with 100% humidity. 3 µL of NV sample was added to each grid, blotted for 1–3 s, and immediately submerged in liquid ethane. Frozen grids were then transferred to a JEOL 3200FS microscope outfitted with a K2 Summit 4k × 4k direct detector (Gatan) and a postcolumn energy filter set to 30 eV. Images were collected at magnifications of 15 000× and 30 000×, with pixel sizes of 2.392 and 1.232 Å, respectively. Images were collected using an exposure time of 1 s and an approximate dose rate of 20e^−^ Å^−2^ s^−1^ per image.

### Characterization of NV Size, Polydispersity Index, Zeta Potential, and Concentration

A Zetasizer system (Malvern Panalytical) was used to determine the size, polydispersity index (PDI), and zeta potential of all synthesized NVs. 500 µL of the sample was diluted 1:100 w/w in 1× PBS and was prepared in polystyrene cuvettes (Bio‐Rad Laboratories) for size and PDI measurements. For each sample, a total of three runs with 10 measurements/run were performed for each sample; the average of these three runs was reported. For the zeta potential measurements, 10 µL of the sample were diluted with 900 µL of MilliQ water and 90 µL of 1× PBS. Prepared samples were transferred to folded capillary disposable cuvettes (Malvern Panalytical). For each measurement, three runs with 15 measurements/run were performed; the average of these three runs was reported. A NanoSight NS300 system (Malvern Panalytical) was used to determine NVs concentration after synthesis. Samples were prepared by diluting NVs in 1× PBS (1:10 000 v/v) and loading them onto the syringe pump. Acquisition settings were the following: screen gain = 1, camera level = 13, flow ratio = 1 mL min^−1^, and temperature = 25 °C. Five measurements of each sample were acquired for each sample, with a duration time of 60 s/sample. A detection threshold of 7 was used to evaluate the final NV concentration.

### Metabolic Assays

For toxicity assays, monolayers of memGFP‐iNeurons were plated at a density of 60 000 cells/well in Matrigel‐coated 96‐well plates. At the time of the MTS assay, cells were incubated with CellTiter 96 Aqueous One Solution Reagent (Promega) in phenol red‐free NM+Dox for 4 h according to the manufacturer's instructions. Absorbance was read at 490 nm using a plate reader (Tecan). Cellular viability was determined by subtracting blank values and normalizing to the control group without NVs. For sphere toxicity assays, iNeuron spheres treated with NVs and incubated with equal volumes of CellTiter‐Glo 3D Reagent (Promega) and phenol red‐free NM+Dox in clear‐bottom, opaque‐walled 96‐well plates. The assay was carried out according to the manufacturer's instructions and the luminescence (RLU) was determined using a plate reader (Tecan). Measured RLU values, which varied linearly with the size of the sphere, were normalized to the cross‐sectional areas of each sphere. Results were then standardized to the mean value of the control groups (untreated, 0 × 10^−6^ m) for both formulations.

### Fluorescence‐Activated Cell Sorting (FACS)

FACS was performed as previously elaborated^[^ ^26^ ^]^ with several modifications. Briefly, monolayers of cells were plated in Matrigel‐coated 24‐well plates at the following densities: day 7 iNeurons, 250 000 cells/well; human astrocytes and microglia, 100 000 cells/well. 24 h before FACS, rhodamine labeled NVs were added to monolayers. Following a 24 h incubation, cells were gently detached with Accutase solution, washed with 1× PBS, centrifuged, and washed again with 1× PBS. Cells were collected into flow cytometry tubes and run on a BD LSRII flow cytometer using the Yel/Grn‐561 nm Laser and the PE filter 585/15 nm.

### Confocal Microscopy

iNeurons or microglia were plated on Matrigel‐coated chamber slides for 48 h before treatment, then incubated with rhodamine labeled NVs for 24 h. iNeurons were then washed 3 times in PBS for 10 min each, then fixed with 4% paraformaldehyde (PFA) for 30 min at 4 °C, rinsed again with PBS 3 times for 10 min each, and mounted with Fluoromount‐G (Southern Biotech) on glass slides. Microglia were fixed, permeabilized with blocking buffer containing 5% (v/v) donkey serum (BioLegend) and 0.25% Triton X‐100 (Sigma‐Aldrich) in PBS at room temperature for 30 min, and then incubated with Iba1 primary antibody (Abcam) overnight at 4 °C. Cells were washed 3 times with PBS, then incubated with secondary blocking buffer (PBS containing 5% donkey serum) and donkey antigoat Alexa Fluor 488 secondary antibody (Thermo Fisher) with 4′,6‐diamidino‐2‐phenylindole (DAPI; Invitrogen) at room temperature for 2 h. After washing again, slides were mounted in Fluoromount‐G (Southern Biotech). After drying, slides were imaged on a DMi8 confocal microscope (Leica) with a 63× oil immersion objective. 3D neural spheres were treated with 500 × 10^−6^ m rhodamine labeled NVs for 24 h in NM+Dox. Spheres were then washed in PBS and fixed with 4% PFA for 45 min at 4 °C. PFA was exchanged with NucBlue Fixed Cell Ready Probes Reagent (Hoechst 33342, Invitrogen) at 2 drops mL^−1^ for 1 h at room temperature. Sphere were cleared in a fructose–glycerol solution^[^ ^65^ ^]^ overnight at room temperature in the dark. Cleared spheres were placed on coverslips and allowed to set overnight. z‐stack images (19–20 µm slices) were acquired on a DMi8 confocal microscope.

### Colocalization Image Analysis

ImageJ (National Institutes of Health) was used to determine colocalization of rhodamine with cellular structures. For images of monolayer cultures, a mask of neuronal cell bodies was constructed by thresholding the mGFP channel to saturation. Using the “Image Calculator” function, the mask was subtracted from the rhodamine (NV) channel. The Raw Integrated Density of the resulting image was recorded using the “Measure” function and divided by the area of the mask in order to produce a measure of colocalization. Mask area was obtained using a histogram of the mask in order to obtain the total number of pixels corresponding to a cell body. To quantify colocalization in spheres, the NucBlue channel was first saturated in order to obtain a mask of the sphere. The mask was then binarized and the number of pixels that comprised the mask was taken to be analogous to the area of the sphere. This mask was also used to exclude all rhodamine (NV) signal that was not colocalized to the sphere, accomplished by subtracting the mask from the rhodamine (NV) channel using the “Image Calculator” function. The Raw Integrated Density of the resulting image was then taken as a measure of the colocalization of NV and neurons within a given sphere and then normalized to total NucBlue signal to control for variations in sphere size.

### Ex Vivo Studies

Whole brains were removed from wild type C57 pups (p4) in accordance with study protocols approved by the Houston Methodist Research Institute Institutional Animal Care and Use Committee (IACUC) and sliced using an NVSLM1 Motorized Advance Vibroslice (World Precision Instruments) in artificial cerebrospinal fluid (ACSF, pH 7.4) containing 1 × 10^−3^ m calcium chloride, 5 × 10^−3^ m magnesium chloride, 10 × 10^−3^ m d‐glucose (all Sigma Aldrich), 4 × 10^−3^ m potassium chloride, 26 × 10^−3^ m sodium bicarbonate, 246 × 10^−3^ m sucrose (all Fisher). Slices were maintained in NM and incubated with 500 × 10^−6^ m NVs for 24 h, washed with PBS, then fixed with 4% PFA for 45 min at 4 °C. Slices were permeabilized with blocking buffer containing 5% (v/v) goat serum (BioLegend) and 0.25% Triton X‐100 (Sigma‐Aldrich) in PBS at room temperature for 30 min, and incubated with primary antibodies including anti‐*β*‐III‐tubulin (TUBB3, BioLegend) and antiglial fibrillary acidic protein (GFAP, Thermo Fisher) overnight at 4 °C. After washing 3 times with PBS, slices were incubated with secondary blocking buffer (PBS containing 5% goat serum) and secondary antibodies, goat antimouse Alexa Fluor 488 or goat antichicken Alexa Fluor plus 647 (both Thermo Fisher) with DAPI at room temperature for 2 h. After washing again, slices were mounted in Fluoromount‐G on glass slides and imaged with DMi8 confocal microscope (Leica) with a 63× oil immersion objective.

### In Vivo Studies

Ten‐week‐old male C57BL/6J mice (24–26 g each) were anesthetized and mounted in a stereotactic frame for trigeminal ganglion (TG) microinjection using the following coordinates: 4.3 mm rostral, 1.5 mm lateral, and 6.24 mm ventral to the lambda.^[^ ^66^ ^]^ 3 µL of each NV formulation was injected to the TG of each mouse (*n* = 3–5 per group). 3 µL of PBS was injected as a control. After 18 h, the animals were euthanized, and TGs were harvested and analyzed with FACS using mouse antineuron‐specific *β*‐III‐tubulin‐APC (1:100; Clone TUJ‐1, R&D systems) or Brilliant Violet 421 anti‐GFAP antibody (1:80; Clone 2E1.E9, BioLegend). Study protocols were approved by the University of Texas MD Anderson Cancer Center Institutional Animal Care and Use Committee (IACUC).

### Statistical Analysis

Outliers were identified and removed using the ROUT method (based on maximum false discovery rate *Q* = 1%) in GraphPad Prism, as noted in figure captions if applicable. Data are presented as mean ± standard errors of the mean (SEM). The sample size (*n*) for every experiment is noted in each figure caption. For FACS analysis, cells in adjacent wells were considered technical replicates, while multiple experiments with independently differentiated cells and independently fabricated NVs represented biological replicates. For in vivo experiments, animals were considered biological replicates. Data from experiments using one biological replicate are averaged across technical replicates, without calculation of significance (e.g., 3D sphere experiments). For experiments with biological replicates, errors were calculated between replicates. Formulations A and B were considered distinct and therefore not statistically compared in any experiments. Significance was determined using two‐tailed unpaired t‐tests (between two groups) or one‐way analysis of variance (ANOVA) followed by Tukey's multiple comparison tests (between multiple groups), as detailed in figure captions. In all figures, *p < 0.05 and ^**^ *p* < 0.01. Prism 9 software (GraphPad) was used for all statistical analysis.

## Conflict of Interest

The authors declare no conflict of interest.

## Author Contributions

A.Z. and C.C. contributed equally to this work. A.Z. and C.C. conceived this research project. A.Z., C.C., F.T., and R.K. designed the experiments. A.Z., M.S., T.N., and G.B. synthesized and characterized the NVs. A.Z., C.C., M.S., M.A. (1), N.B., and R.K. performed the in vitro experiments. A.Z., C.C., T.N., J.C., M.A. (2), and T.X. performed the in vivo experiments. A.Z., C.C., M.S., and A.S. analyzed the data. A.Z., C.C., M.S., F.T., and R.K. wrote and edited the manuscript. M.A. (2), E.T., F.T., and R.K. provided resources, funding, and supervision.

## Supporting information

Supporting Information

## Data Availability

Research data are not shared.
