# Supplementary material for: Humanized Biomimetic Nanovesicles for Neuron Targeting
Source: Adv Sci (Weinh). 2021 Aug 11;8(19):2101437. doi: 10.1002/advs.202101437 (PMC8498895; doi:10.1002/advs.202101437)
Supplement: Supplementary file 1 — Supporting Information [file ADVS-8-2101437-s001.pdf]

WILEY-VCH

© 2021 Wiley-VCH GmbH

ADVANCED SCIENCE
Open Access

Supporting Information

for *Adv. Sci.,* DOI: 10.1002/advs202101437

Humanized Biomimetic Nanovesicles for Neuron Targeting

Assaf Zinger, Caroline Cvetkovic, Manuela Sushnitha, Tomoyuki Naoi, Gherardo Baudo, Morgan Anderson, Arya Shetty, Nupur Basu, Jennifer Covello, Ennio Tasciotti, Moran Amit, Tongxin Xie, Francesca Taraballi *, and Robert Krencik **

Supporting Information

Humanized Biomimetic Nanovesicles for Neuron Targeting

Assaf Zinger [†,]*** *Caroline Cvetkovic* [†]*, Manuela Sushnitha, Tomoyuki Naoi, Gherardo Baudo, Morgan Anderson, Arya Shetty, Nupur Basu, Jennifer Covello, Ennio Tasciotti, Moran Amit, Tongxin Xie, Francesca Taraballi *, and Robert Krencik **

Included in this section are the following items:
Figure S1. Neural NV synthesis and biological and physiochemical characterization.
Figure S2. Cellular association of NVs with iNeurons *in vitro*.
Figure S3. Gating strategy and normalization for flow cytometry analysis of NV association with iNeurons.
Figure S4. NV association with other neural cell types and potential neuronal association mechanism.
Figure S5. *Ex vivo* association of NVs with murine brain slices.
Figure S6. *In vivo* NV association via trigeminal ganglion injection.
Table S1. Lipid composition of Formulations A and B.

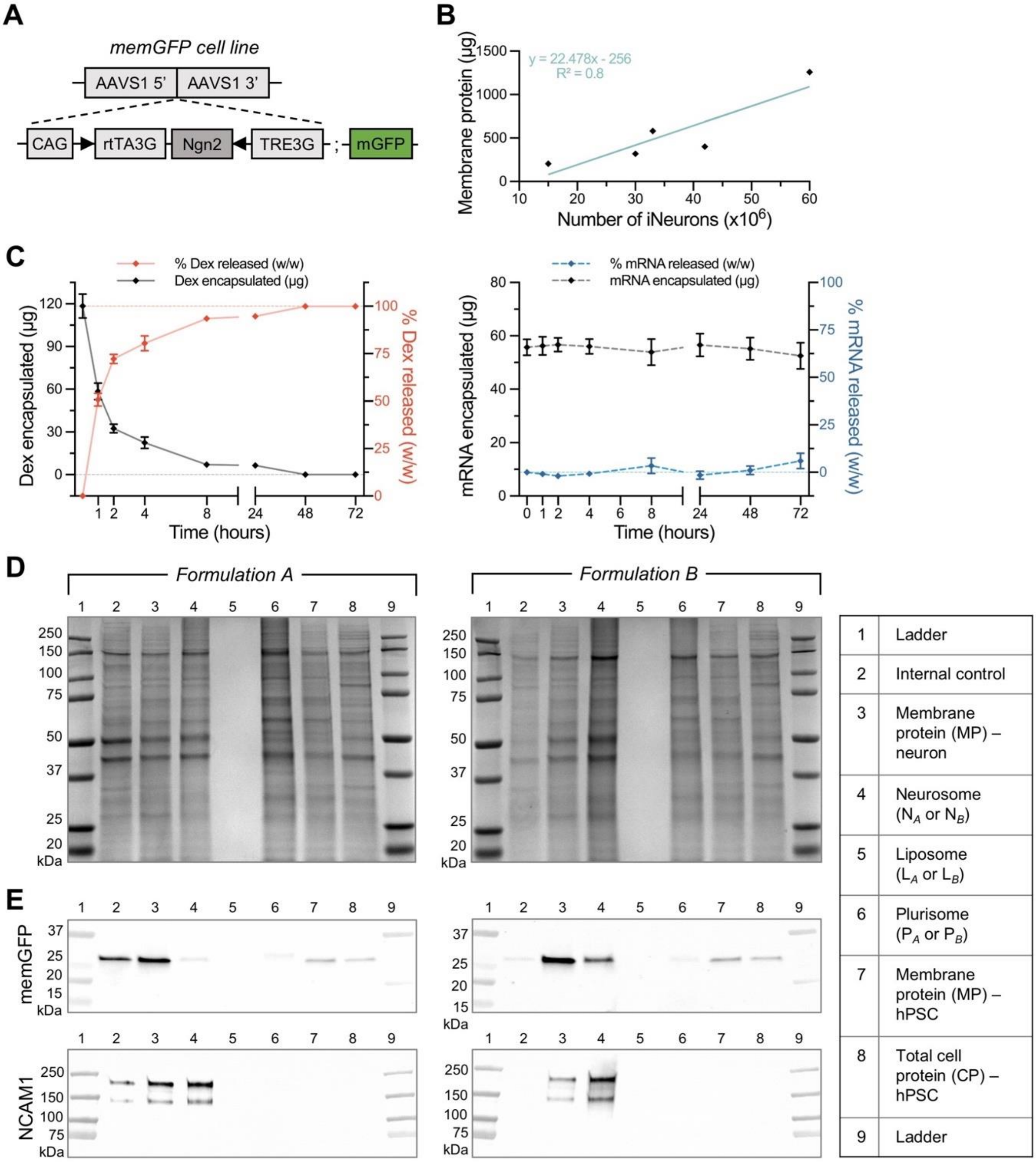

F

	Formulation A			*Formulation B*		
	Liposome (L_A)	Plurisome (P_A)	Neurosome (N_A)	Liposome (L_B)	Plurisome (P_B)	Neurosome (N_B)
Size (nm)	117.5 ± 5.7 (n = 4)	121.3 ± 2.6 (n = 3)	116.3 ± 1.8 (n = 4)	111.5 ± 2.8 (n = 6)	124.7 ± 3.4 (n = 3)	115.6 ± 3.9 (n = 7)
PDI	0.13 ± 0.019 (n = 4)	0.12 ± 0.008 (n = 3)	0.11 ± 0.004 (n = 4)	0.07 ± 0.012 (n = 6)	0.07 ± 0.009 (n = 3)	0.10 ± 0.013 (n = 7)
Zeta potential (mV)	-6.0 ± 1.3 (n = 4)	-9.0 ± 0.6 (n = 3)	-8.7 ± 1.0 (n = 4)	-1.7 ± 0.3 (n = 6)	-2.7 ± 0.7 (n = 3)	-2.2 ± 0.4 (n = 7)

Figure S1. Neural NV synthesis and biological and physiochemical characterization. (A) Targeting scheme used to generate membrane-bound green fluorescent protein (memGFP)-containing, dox-inducible iNeurons. **(B)** Membrane proteins (MPs, plotted as a function of starting

cell density in millions of iNeurons) were extracted using a ProteoExtract® Native Membrane Protein Extraction kit and quantified using a Pierce Rapid Gold BCA kit. iNeurons provided an average of 14.5±2.1 µg of MP per million cells. **(C)** Drug release profiles for dexamethasone (Dex; *left*) encapsulated in L_A NVs (n=4 independent NV batches) and CleanCap® FLuc mRNA 5moU (*right*) encapsulated in L_B NVs (n=3 independent NV batches) over 72 h. Left axes show Dex or mRNA encapsulated in NVs (µg) and right axes show % (w/w) released from NVs. Results are shown as mean ± SEM. **(D)** Coomassie blue staining after SDS-PAGE separation confirmed the transfer of proteins from cell lysate to membrane fraction into NVs. Column labels are shown on the right. **(E)** Immunoblotting of GFP and NCAM1 proteins. (Bands are replicated in Figure 1D, where lanes 7, 6, 3, and 4 are shown left to right, respectively, for both formulations A and B. Dividing lines between lanes 7 and 6 indicate splicing and rearrangement from original image.) **(F)** Detailed results (mean values ± SEM, with sample size noted) of physiochemical characterization of NVs from formulations A and B, including NV size, polydispersity index (PDI), and zeta potential, as measured using a Zetasizer Nano ZS system (see Figure 1F).

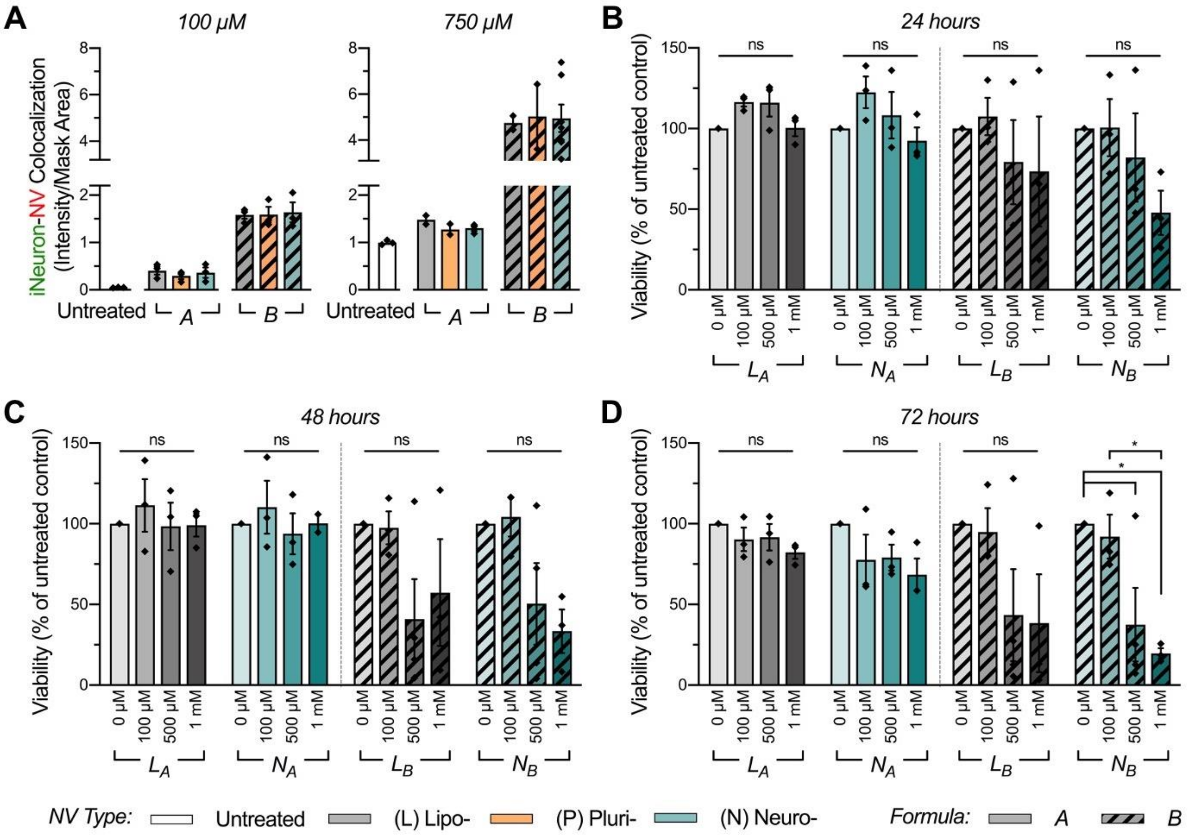

Figure S2. Cellular association of NVs with iNeurons *in vitro*. (A) Colocalization of 100 µM (*left*) and 750 µM (*right*) rhodamine labeled NVs with mGFP-positive iNeurons after 24 h incubation (n=2-7 images per group; see images from 750 µM incubation in Figure 2A). **(B-D)** Extended cellular viability results for **(B)** 24 h, **(C)** 48 h, and **(D)** 72 h incubation periods at concentrations ranging from 100 µM to 1 mM (normalized to untreated cells; n=3-4 independent batches of cells and NVs per group). For Figure S2A-D, results are shown as mean ± SEM. For viability experiments (Figures S2B-D), one-way ANOVA followed by Tukey's multiple comparison test was used to determine statistical probabilities between concentrations of NVs within the same formulation and NV type, for multiple incubation times, with *$p<0.05$.

A

B

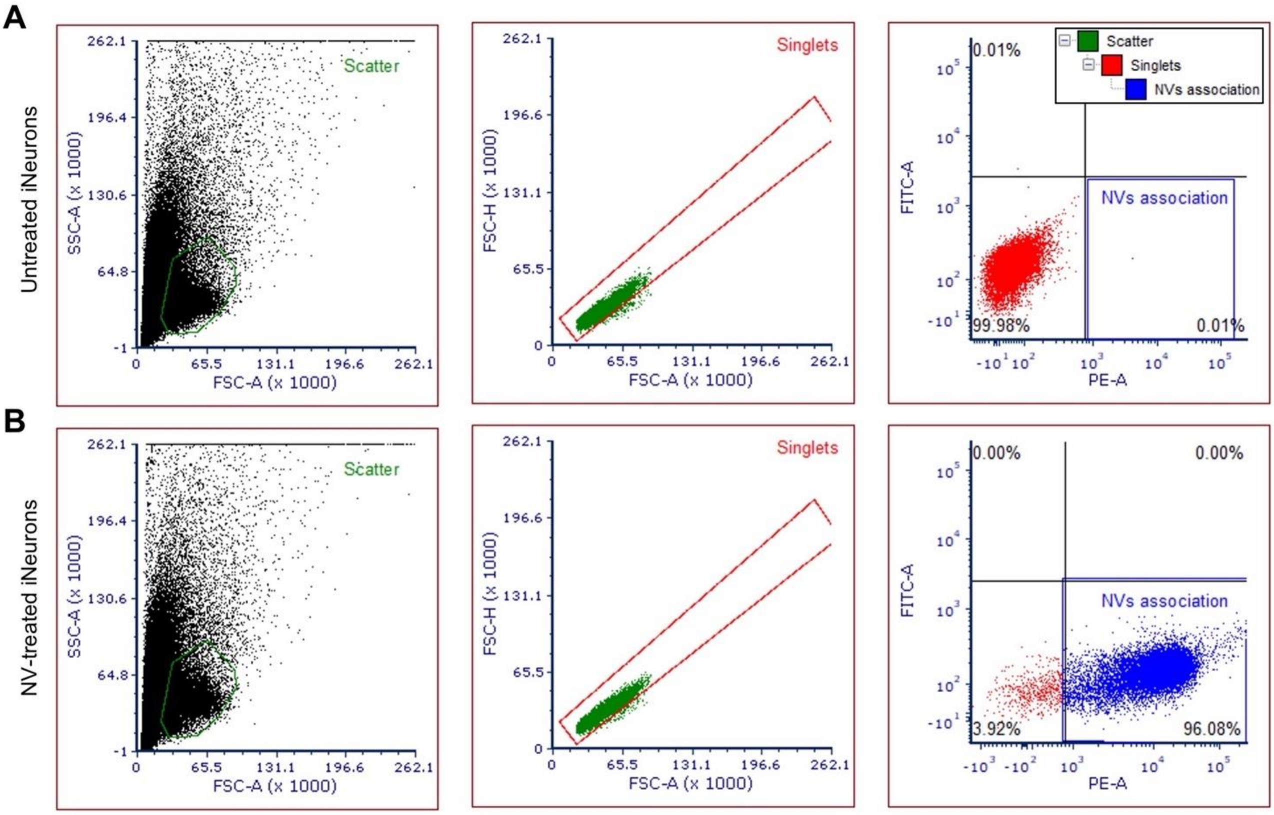

C

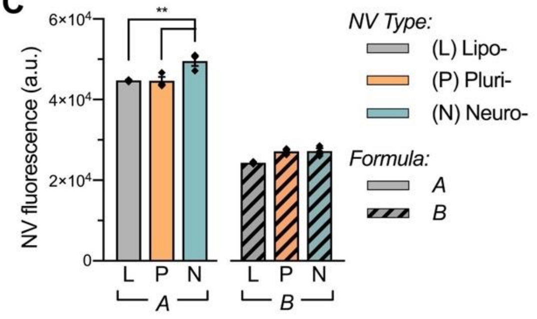

D

	Formulation A		
	Liposome (L_A)	Plurisome (P_A)	Neurosome (N_A)
Normalized fluorescent intensity (a.u.)	1.0 ± 0.0 (n = 4)	1.04 ± 0.12 (n = 3)	1.4 ± 0.08 (n = 4)

	Formulation B		
	Liposome (L_B)	Plurisome (P_B)	Neurosome (N_B)
Normalized fluorescent intensity (a.u.)	7.83 ± 1.29 (n = 6)	7.07 ± 0.83 (n = 5)	7.16 ± 1.01 (n = 6)

Figure S3. Gating strategy and normalization for flow cytometry analysis of NV association with iNeurons. (A) Gating for the scatter, singlets, and NV association with untreated iNeurons. **(B)** Gating for the scatter, singlets, and NV association with NV-treated iNeurons. **(C)** Fluorescent intensity (arbitrary units, a.u.) of NVs from formulations A and B, measured by a microplate reader before treatment to cells (n=3 independent batches of NVs per group). **(D)** Detailed results (mean values ± SEM, with sample size noted) of normalized median fluorescent intensity from quantitative fluorescence-activated cell sorting (FACS) to quantify NV association with iNeurons (see Figure 2C). For Figure S3C, results are shown as mean ± SEM. One-way ANOVA followed by Tukey's multiple comparison test was used to determine statistical probabilities from fluorescence intensity experiments, within each formulation, with *p<0.05, **p<0.01.

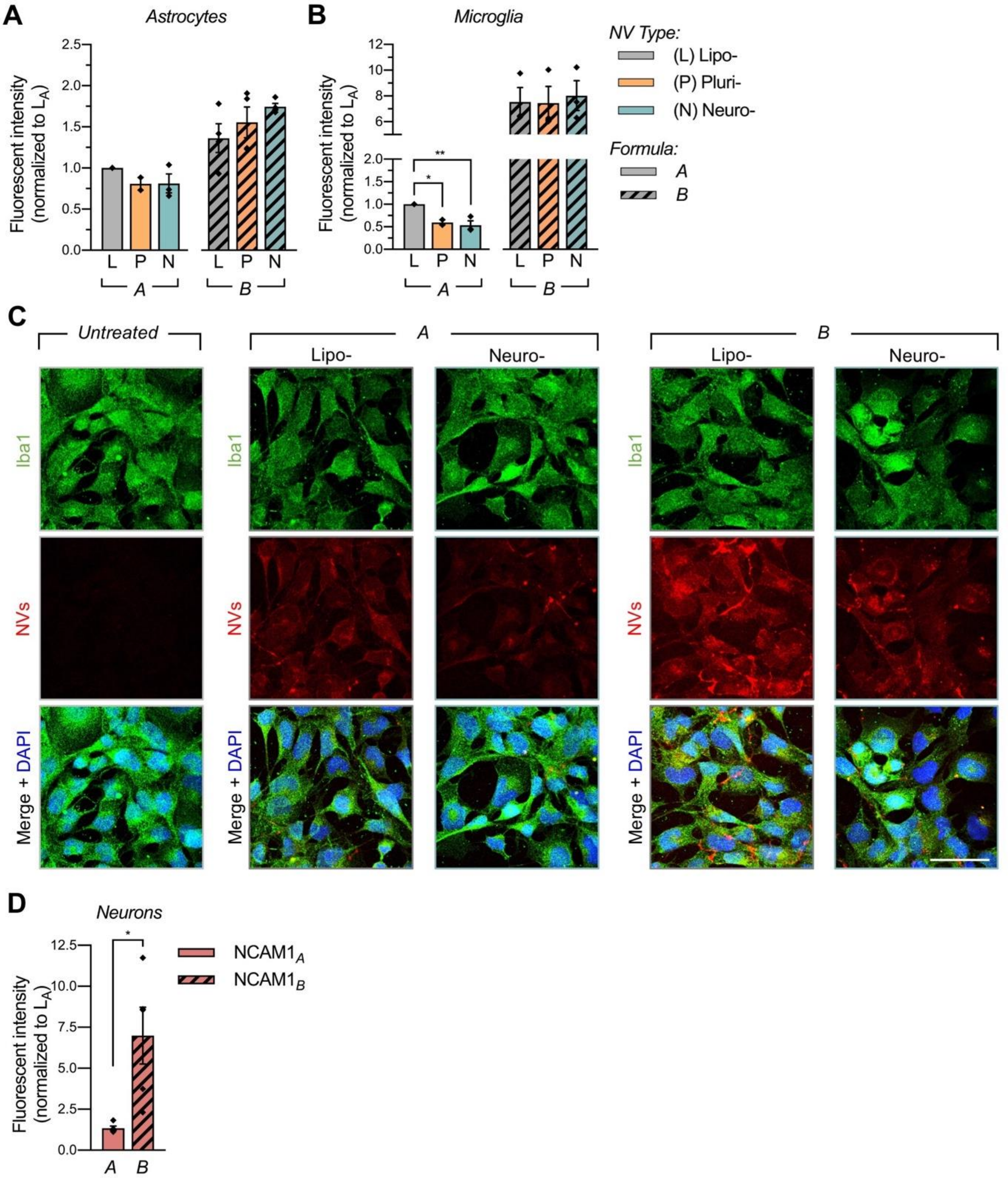

Figure S4. NV association with other neural cell types and potential neuronal association mechanism. (A-B) Monolayers of human **(A)** astrocytes and **(B)** HMC3 microglia were incubated with 100 µM rhodamine labeled NVs for 24 h to quantitatively assess association of liposomes, plurisomes, and neurosomes of formulations A and B using high throughput fluorescence-activated cell sorting (FACS). No significant differences in cellular association were observed between NV types of the same lipid formulation for astrocytes (n=2-7 independent batches of cells and NVs). While no significant differences in cellular association was observed between NV types of formulation B, a significant decrease in cellular association was observed in P_A and N_A compared to L_A in microglia cells (n=2-3 independent batches of cells and NVs). Data are presented as median fluorescent intensities normalized to L_A for each cell type. **(C)** HMC3 were incubated with 100 µM rhodamine labeled liposomes and neurosomes from both formulations A and B for 24 h

and stained for Iba1. Scale: 50 µm. **(D)** The potential of NCAM1 as a targeting mechanism for neuronal association was examined by fabricating NVs composed only from NCAM1 membrane protein in place of total membrane protein. Human recombinant NCAM1 protein was integrated into NV lipid bilayers from lipid formulations A and B to generate $NCAM1_A$ and $NCAM1_B$ NVs. iNeurons were incubated with $NCAM1_A$ and $NCAM1_B$ for 24 h and evaluated for NV association. Association was quantified using FACS (n=5 independent batches of cells and NVs). For Figures S4A, S4B, and S4D, results are shown as mean ± SEM. For Figures S4A and S4B, one-way ANOVA followed by Tukey's multiple comparison test was used to determine statistical probabilities between NVs of different protein sources, within each formulation, with *$p<0.05$. For Figure S4D, significance was determined using a two-tailed unpaired t-test between $NCAM1_A$ and $NCAM1_B$, with *$p<0.05$.

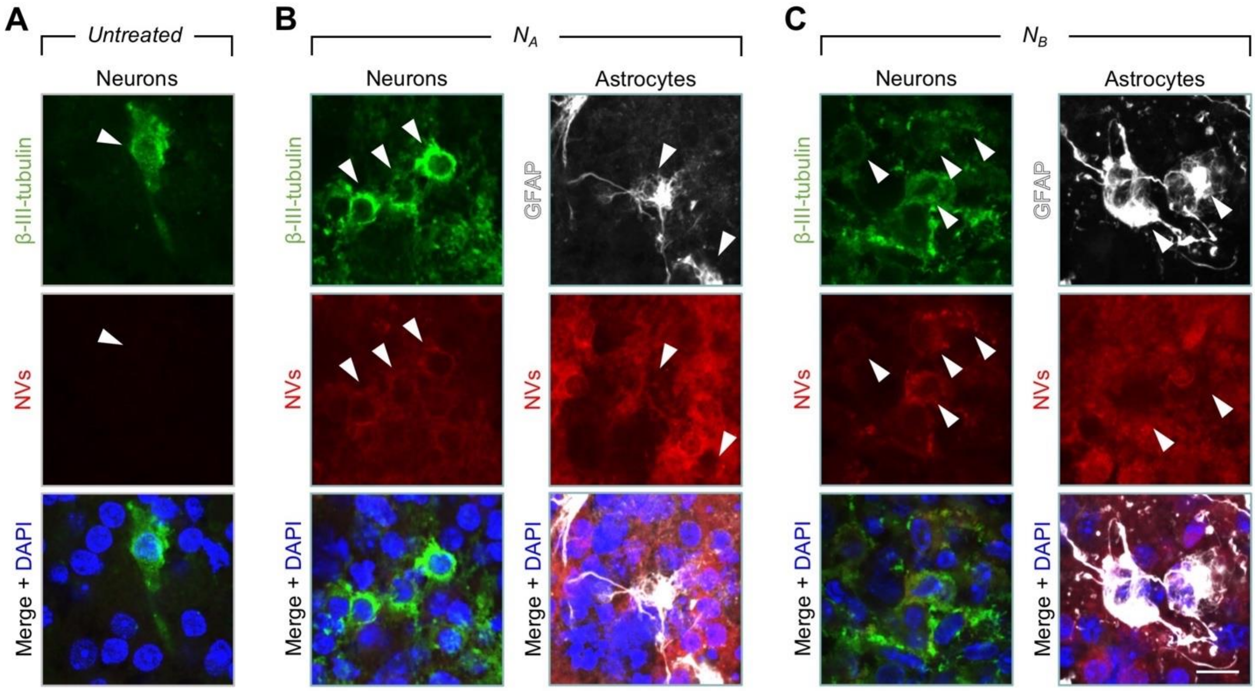

Figure S5. *Ex vivo* association of NVs with murine brain slices. Brain slices from p4 pups were incubated with 500 µM rhodamine labeled neurosomes for 24 h and stained for neuronal (β-III-tubulin) and astrocyte (glial fibrillary acidic protein, GFAP) cell-restricted markers. Shown are **(A)** untreated, **(B)** N_A-treated, and **(C)** N_B-treated slices with regions of neurons or astrocytes. White arrows indicate cell bodies. Scale: 10 µm.

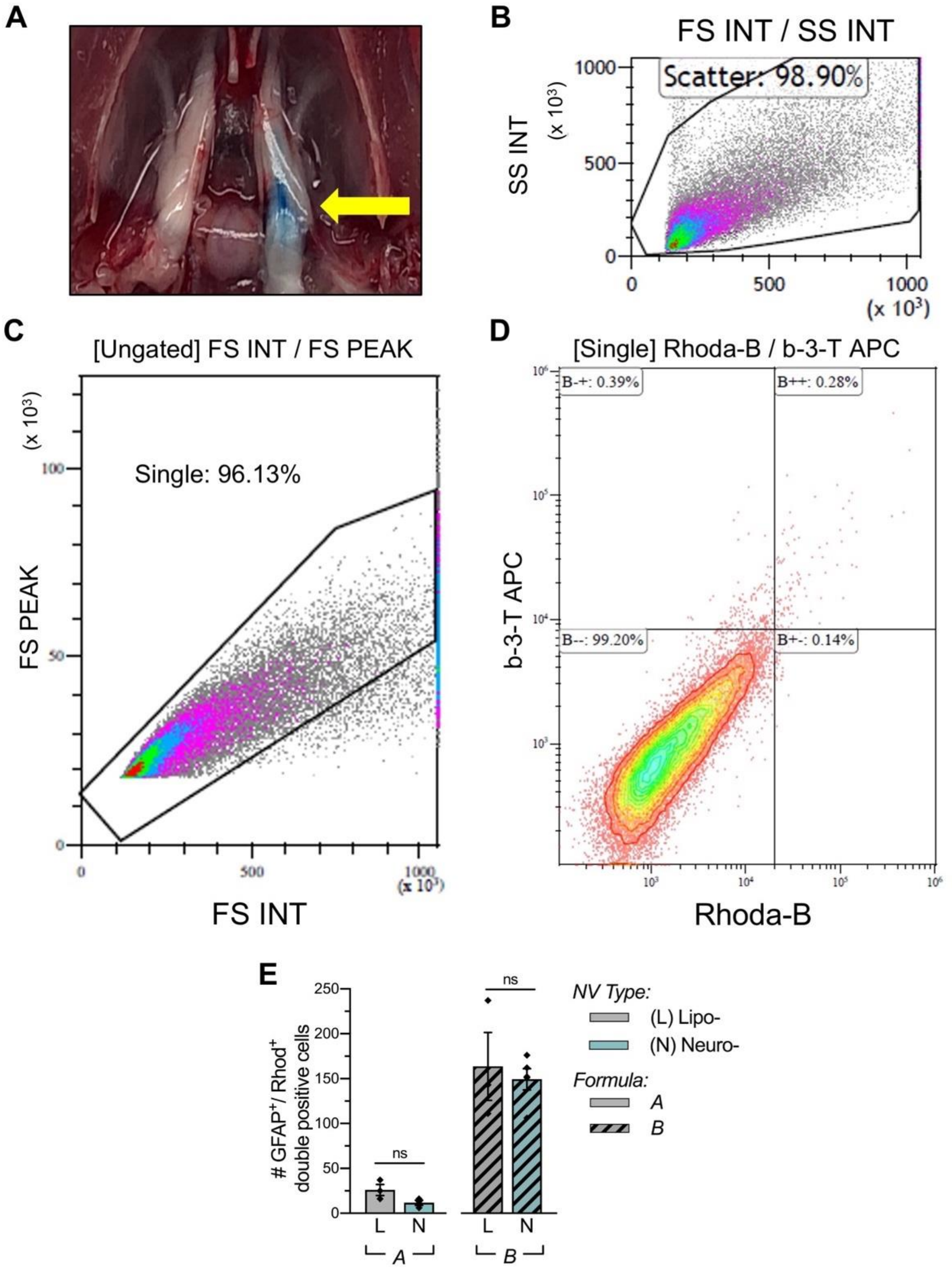

Figure S6. ***In vivo* NV association via trigeminal ganglion injection. (A)** Coomassie blue (marked by the yellow arrow) was injected to demonstrate the site of injection for the NVs. Flow cytometry gating strategy for the **(B)** scatter and **(C)** single cells from an untreated mouse. **(D)** Gating for β-III-tubulin (b-3-T) positive neurons (see Figure 3F). **(E)** FACS analysis for NV association with astrocytes indicated similar levels of association between both NV types, as assessed by double-positive signal of rhodamine (NVs) with fluorescently labeled GFAP (n=3-5 mice per group of NVs). For Figure S6E, results are shown as mean ± SEM. Significance was determined using a two-tailed unpaired t-test between neurosomes and liposomes for formulations A and B for *in vivo* FACS experiments, with *$p<0.05$.

Formulation	Lipids		Molar Ratio
A	DPPC	Dipalmitoylphosphatidylcholine	4
	DOPC	1,2-dioleoyl-sn-glycero-3-phosphocholine	3
	Cholesterol		3
B	16:0 DAP	16:0 1,2-dipalmitoyl-3-dimethylammonium-propane	4.2
	DSPE-PEG2000	1,2-distearoyl-sn-glycero-3-phosphoethanolamine N-[carboxy(polyethylene glycol)-2000]	1
	Cholesterol		4.8

Table S1. Lipid composition of Formulations A and B.
